# Direct-Acting Oral Anticoagulants and Potential Inconsistencies with FDA-Approved Dosing for Non-Valvular Atrial Fibrillation: A Retrospective Real-World Analysis Across Nine US Healthcare Systems

**DOI:** 10.1007/s11606-024-09106-w

**Published:** 2024-10-18

**Authors:** Bonnie DeLor, Jon J. Glover, Timothy J. Hartman, Laura L. Manzey, Mohammad Ateya, Shelby Kelsh, Katie Taylor, Wesley R. Zemrak, Jaclynne R. Gowen, Ann Parks, Carmen Gust, Charles Medico, Ukwen C. Akpoji, Shane Naylor, Carolyn W. Chou, Gregory Fakelmann, Sara Hart, Eryne E. Wiethorn, Thach Trinh, William W. Wilson, Rachel Bowen, Jennifer Stanton, Laura Duvall, Lynette T. Davis, for the D-DARE Consortium, Alexander Ansara, Ashley Child, Richard W. Dettloff, Saira Naim Haqu, Saba Sarsam, Heather Somand, Christina Wadsworth

**Affiliations:** 1 Corewell Health/Ferris State University, Grand Rapids, MI USA; 2 MaineHealth, Portland, ME USA; 3 Mass General Brigham - Wentworth Health Partners, Dover, NH USA; 4 ThedaCare, Neenah, WI USA; 5 MetroHealth, Cleveland, OH USA; 6 Pfizer Inc, 66 Hudson Blvd E, New York, NY 10001 USA; 7 Baptist Health, Louisville, KY USA; 8 Indiana University Health, Indianapolis, IN USA; 9 Parkview Health, Fort Wayne, IN USA; 10 Premier Health, Dayton, OH USA

**Keywords:** atrial fibrillation, stroke prevention, anticoagulation, stewardship, dosing

## Abstract

**Background:**

Direct-acting oral anticoagulants (DOACs) are recommended to reduce risk of stroke and systemic embolism in patients with non-valvular atrial fibrillation (NVAF). However, DOAC dosing inconsistent with FDA-approved product labels is common and associated with poor clinical outcomes.

**Objectives:**

Identify DOAC dosing inconsistent with FDA-approved product labels in ambulatory care patients with NVAF; identify variables associated with dosing lower and higher than label.

**Design:**

Retrospective analysis using electronic health records from nine US healthcare systems.

**Patients:**

Adults with NVAF receiving DOAC therapy in 2022.

**Main Measures:**

Rates of label-inconsistent dosing; multivariable regression analysis to identify demographic and clinical variables associated with dosing lower and higher than label.

**Key Results:**

Among 51,128 NVAF patients (56.1% male, 94.3% White, mean [SD] age 73.5 [10.5] years), 5008 (9.8%) were prescribed label-inconsistent doses of DOACs (6.8% lower and 3.0% higher than label). Age ≥ 75 years, renal impairment, and hypertension were significantly associated with inconsistent dosing both higher and lower than label. Female sex and higher weight were significantly associated with dosing lower than label, as were heart failure, vascular or liver disease, and bleeding history. Dosing higher than label was significantly associated with male sex, race (African American/Black), weight < 60 kg, and use of drugs with potential drug-drug interactions. When prescribed by primary care physicians, DOAC doses were 37% (95% CI, 27–49%) more likely to be lower than label and 30% (95% CI, 16–46%) more likely to be higher than label than when prescribed by cardiologists or electrophysiologists. Label-inconsistent dosing varied (6.7 to 15.8%) across participating systems.

**Conclusions:**

DOAC dosing inconsistent with label varied by demographics, clinical characteristics, prescriber specialty, and healthcare system, suggesting a need to monitor and assess dosing decisions in NVAF. Identification of variables associated with dosing inconsistencies may enable targeted interventions to ensure label-consistent dosing in vulnerable populations.

**Supplementary Information:**

The online version contains supplementary material available at 10.1007/s11606-024-09106-w.

## INTRODUCTION

The benefit of direct-acting oral anticoagulants (DOACs) to reduce the risk of stroke in patients with atrial fibrillation in the absence of moderate to severe mitral stenosis or prosthetic heart valves, previously referred to as non-valvular atrial fibrillation (NVAF), is well-established when therapy is based on risk stratification; dosing is consistent with the United States (US) Food and Drug Administration (FDA)–approved product label; and when the patient is adherent to therapy.^1–3^ DOACs, including apixaban, dabigatran, edoxaban, and rivaroxaban, provide alternatives to warfarin that do not require laboratory monitoring of the international normalized ratio (INR). These DOACs demonstrated efficacy at least similar to warfarin with improved safety profiles,^4–8^ and are recommended as first-line treatment to reduce the risk of stroke and systemic embolism in patients with NVAF.^2,3^

All anticoagulation therapy is associated with bleeding risk.^9–11^ Patient safety initiatives to promote quality care for anticoagulant use have been developed to reduce this risk.^12,13^ As DOAC dosing regimens vary by agent and indication, they require consideration of demographic and clinical variables for individualized dose adjustment, increasing dosing complexity. While FDA-approved medication labels specifically indicate when and how to adjust doses,^14–17^ label-inconsistent dosing of DOACs is common,^18–30^ and meta-analyses suggest associations between label-inconsistent dosing and risk of adverse patient outcomes.^31–39^ Despite variability in outcomes among these meta-analyses, dosing lower than label was generally associated with higher risks of all-cause mortality^31,32,35,37,39^ and stroke/systemic embolism^31–34,39^ but not lower bleeding risk.^32–35,37–39^ Dosing higher than label, however, was associated with increased risks of bleeding and stroke/systemic embolism.^31–33,37^

Depending on country and healthcare setting, up to 29% of patients may receive doses inconsistent with approved labeling. While the rate of label-inconsistent dosing varies among DOACs, doses lower than label are more frequently prescribed than doses higher than label.^18–30^ However, there is only limited evidence from recent, large, real-world studies on dose-inconsistency in the US and the factors that may drive differences in dosing. One such study of patients with NVAF treated with DOACs reported that label-inconsistent dosing (12.6% underdosed and 5.0% overdosed) was associated with lower treatment adherence and was more frequent with worsening renal impairment.^29^ While that study highlighted the need to improve DOAC dosing through quality improvement initiatives and characterized factors associated with label-inconsistent dosing, it encompassed early DOAC prescribing (2015 to 2017) and relied on medical and pharmacy claims data rather than electronic health records (EHRs). In contrast to claims data, which reflect administrative data, EHRs are more inclusive of clinical information, enabling analysis of clinical variables potentially associated with dosing patterns.

The primary objective of this multi-site study was to use EHR data to characterize low and high DOAC dosing inconsistent with FDA-approved labeling. A secondary objective was to use these data to identify variables associated with low and high label-inconsistent dosing, with the goal of providing actionable information for improving care in patients with NVAF treated with DOACs.

## METHODS

### Study Design and Population

Data for this retrospective, cross-sectional, real-world study were extracted from EHRs of nine large US healthcare systems across the Midwest (*N* = 7) and Northeast regions (*N* = 2). Participating systems used a standardized data extraction guide (including data dictionary and code book) to obtain the data, which were extracted using Structured Query Language (SQL) from the Medication, Demographic, Encounter, Vital, and Laboratory EHR relational database tables. Patient identifiers were removed prior to sharing the data securely with the central study team. Initial data files were reviewed by clinicians at each healthcare system and by the central study team; discrepancies or issues were resolved prior to finalizing data release. The study was approved by a central institutional review board (Advarra, Columbia, MD), and local site institutional review board approval processes were followed. The study was compliant with the Health Insurance Portability and Accountability Act (HIPAA).

The evaluated population consisted of adult patients who had EHR documentation of NVAF and were receiving a DOAC as determined by an outpatient-mode prescription order between January 1, 2022, and December 31, 2022, regardless of the encounter setting. The date of the most recent prescription order in the EHR was defined as the index date. For inclusion, patients ≥ 18 years old on the index date were required to have a confirmed diagnosis of atrial fibrillation or atrial flutter based on International Classification of Diseases 10th Revision Clinical Modification (ICD-10-CM) codes (Supplemental Table 1) and were prescribed a DOAC including apixaban, dabigatran, edoxaban, or rivaroxaban. These medications were identified via FDB® generic sequence numbers or Medispan® 14-digit generic product identifiers (GPI) and/or American Hospital Formulary Service® classes depending on the medication information source used by the healthcare system. To comply with HIPAA, patients > 89 years old were coded as “90” years old. Patients were excluded if they had valvular heart disease, defined by history of mechanical valve; rheumatic mitral valvular heart disease; heart valve replacement/transplant in the 12-month pre-index period; or received anticoagulation for any indication other than NVAF, including history of hip/knee replacement 6 weeks pre-index or history of venous thromboembolism 12 months pre-index. Pregnancy or childbirth within the study period resulted in exclusion. Patients were also excluded from the analysis if they had a combination of reasons for the drug not being indicated (e.g., use of drugs with known interactions in addition to renal impairment), or if they were missing parameters needed for dosing, such as renal function, weight, or sufficient information to establish daily dosing (e.g., days’ supply, quantity dispensed, or administration frequency).

### Outcomes and Variables

Rates of FDA label–consistent and label-inconsistent dosing were determined, focusing on dosing lower and higher than label and variables associated with these patterns. Product information labels for apixaban,^14^ dabigatran,^15^ edoxaban,^16^ and rivaroxaban^17^ were used to derive the FDA-approved dosing (Supplemental Table 2). To evaluate whether the dose prescribed on the index date was consistent or inconsistent with FDA-approved label, the daily dose was determined by strength of the dosage form and frequency of administration or, when missing, quantity dispensed divided by days supplied. The calculated daily dose was then compared to the label dose. Label-consistent dosing categories were defined as full dose when the daily dose was equal to the label dose, and reduced dose when the dose was reduced according to label for creatinine clearance (CrCl), hemodialysis, and drug-drug interactions (DDIs). Label-inconsistent doses were categorized as lower or higher than label when they were less than or greater than label indication, respectively, based on patient characteristics from available EHR data.

Dosing patterns were characterized based on patient demographic and clinical variables at baseline (i.e., index date). Demographic variables were age, sex, and race/ethnicity. Clinical variables included weight and renal function based on CrCl, which was calculated using the Cockcroft-Gault equation^40^ and actual body weight instead of ideal body weight for consistency with DOAC clinical trials. Other clinical variables were the presence of specific comorbid conditions identified using ICD-10-CM codes including heart failure (HF), hypertension, diabetes, vascular disease, and stroke or transient ischemic attack (TIA). Stroke and bleeding risks were based on CHA_2_DS_2_-VASc^41,42^ and HAS-BLED^43^ scores, respectively, and were calculated by aggregating points of the score components. Patients were considered to have a DDI if they had a prescription within the 3-month pre-index period for a medication listed in the FDA label as requiring a dose adjustment, including dronedarone, itraconazole, ketoconazole, and ritonavir-containing products. Prescribing clinicians were categorized as primary care providers (PCP), cardiologists/electrophysiologists (EP), or other/unknown.

### Statistical Analysis

Analysis and reporting of results are consistent with the STROBE reporting guidelines and checklist for cross-sectional observational studies.^44^ Demographic and clinical characteristics are reported using descriptive statistics with frequency (percent) for categorical variables and mean and standard deviation (SD) or median and interquartile range (IQR) for continuous variables. Populations prescribed doses lower or higher than label were each compared with the label-consistent population using Student’s *t*-test for continuous variables and chi-square, Fisher’s exact, and/or two-proportion tests for categorial variables, as appropriate. Label-consistent/inconsistent dosing was evaluated for individual DOACs for the aggregate cohort and stratified by CrCl categories (≥ 60 ml/min, 30–59 ml/min, and < 30 ml/min). Multivariable logistic regression with backwards elimination (threshold of *P* < 0.05 for variable retention) was used to identify variables associated with dosing lower and higher than label. Results of the models, adjusted for demographic and clinical covariates, are presented as adjusted odds ratios (aOR) with 95% confidence intervals (CI). Overall difference in label-inconsistent dosing among the healthcare systems was also evaluated. Statistical analyses were conducted using StataCorp 2017 (*Stata Statistical Software:* Release 15, College Station, TX) with two-tailed alpha = 0.05.

## RESULTS

### Site Characteristics and Patient Population

Six of the nine participating healthcare systems had academic affiliations, eight sites reported having a DOAC dosing quality initiative, and five had anticoagulation stewardship programs (Supplemental Table 3). Among these systems, 64,238 patients were identified with an index DOAC in the initial data set, of whom 5877 (9.1%) were excluded for lacking a diagnosis code for atrial fibrillation or atrial flutter or met other diagnosis- or procedure-based exclusion criteria. Another 7233 patients (11.3%) were excluded for missing weight and/or serum creatinine (Scr) levels, resulting in a final cohort of 51,128 patients.

The population was 56.1% male, 94.3% White, with a mean (SD) age of 73.5 (10.5) years; 49.8% were ≥ 75 years old (Table 1). Patients generally had a high risk of stroke (median [IQR)] CHA_2_DS_2_-VASc score = 3 [3]) and a moderate-to-high bleeding risk (median [IQR] HAS-BLED score = 2 [2]). Among the evaluated comorbidities, hypertension was the most frequent (61.7%), and 15.6% had a history of stroke or TIA; use of medications having DDIs with DOACs was 0.7%.

**Table 1:** Demographic and Clinical Characteristics

Variable	All patients *N* = 51,128	Label-consistent dose *N* = 46,120	Label-inconsistent dose			
			**Lower than label** ***N* = 3454**	***P* vs label-consistent dose**	**Higher than label** ***N* = 1554**	***P* vs label-consistent dose**
Age, years, mean (SD)	73.5 (10.5)	72.8 (10.4)	78.8 (9.8)	< 0.001	80.9 (7.4)	< 0.001
Age categories, years, *n* (%)				< 0.001		< 0.001
< 65	9284 (18.2)	8907 (19.3)	311 (9.0)		67 (4.3)	
65 to 74	16,388 (32.1)	15,585 (33.8)	638 (18.5)		165 (10.6)	
≥ 75	25,456 (49.8)	21,629 (46.9)	2505 (72.5)		1322 (85.1)	
Sex, *n* (%)				< 0.001		< 0.001
Female	22,453 (43.9)	19,720 (42.8)	1886 (54.6)		847 (54.5)	
Male	28,675 (56.1)	26,400 (57.2)	1568 (45.4)		707 (45.5)	
Race, *n* (%)				0.819		< 0.001
White	48,219 (94.3)	43,528 (94.4)	3258 (94.3)		1433 (92.2)	
African American/Black	1946 (3.8)	1727 (3.7)	130 (3.8)		89 (5.7)	
Asian	223 (0.4)	201 (0.4)	12 (0.4)		10 (0.6)	
Other/unknown	740 (1.5)	664 (1.4)	54 (1.6)		22 (1.4)	
Ethnicity (Hispanic), *n* (%)	514 (1.0)	461 (1.0)	32 (0.9)	0.727	21 (1.4)	0.388
CHA_2_DS_2_-VASc, median (IQR)	3 (3)	3 (2)	4 (2)	< 0.001	4 (2)	< 0.001
CHA_2_DS_2_-VASc components, *n* (%)						
Heart failure	14,603 (28.6)	12,791 (27.7)	1287 (37.3)	< 0.001	525 (33.8)	< 0.001
Hypertension	31,549 (61.7)	27,975 (60.7)	2477 (71.7)	< 0.001	1097 (70.6)	< 0.001
Diabetes	14,388 (28.1)	12,879 (27.9)	1099 (37.8)	< 0.001	410 (26.4)	0.183
Stroke/TIA	7954 (15.6)	7056 (15.3)	616 (17.8)	< 0.001	282 (18.2)	0.002
Vascular disease	13,176 (25.8)	11,666 (25.3)	1076 (31.2)	< 0.001	434 (27.9)	0.019
HAS-BLED, median (IQR)	2 (2)	2 (2)	3 (2)	< 0.001	3 (2)	< 0.001
HAS-BLED components, *n* (%)						
Renal	9014 (17.6)	7545 (16.4)	1045 (30.3)	< 0.001	424 (27.3)	< 0.001
Liver	2717 (5.3)	2420 (5.3)	231 (6.7)	< 0.001	66 (4.3)	0.081
Prior bleed	15,451 (30.2)	13,391 (29.0)	1470 (42.6)	< 0.001	590 (38.0)	< 0.001
Alcohol use	1570 (3.1)	1448 (3.1)	92 (2.7)	0.120	30 (1.9)	0.007
Medications (e.g., NSAIDs)	17,490 (34.2)	15,705 (34.1)	1271 (36.8)	0.001	514 (33.1)	0.424
CrCl (ml/min), mean (SD)	83.6 (41.6)	85.3 (41.7)	61.1 (32.3)	< 0.001	47.6 (24.1)	< 0.001
Hemodialysis, *n* (%)	112 (0.2)	78 (0.2)	21 (0.6)	< 0.001	13 (0.8)	< 0.001
Weight, kg, mean (SD)	91.7 (25.2)	92.8 (25.2)	84 (22.8)	< 0.001	75.4 (21.3)	< 0.001
Weight categories, kg, *n* (%)				< 0.001		< 0.001
< 60	3911 (7.7)	3215 (7.0)	226 (6.5)		470 (30.2)	
60–120	40,907 (80.0)	36,892 (80.0)	2988 (86.5)		1027 (66.1)	
> 120	6310 (12.3)	6013 (13.0)	240 (7.0)		57 (3.7)	
Drug-drug interactions, *n* (%)	337 (0.7)	208 (0.5)	9 (0.3)	0.102	120 (7.7)	< 0.001
Clinician specialty, *n* (%)				< 0.001		< 0.001
Cardiology/EP	24,375 (47.7)	22,467 (48.7)	1273 (36.9)		635 (40.9)	
Primary care	21,003 (41.1)	18,706 (40.6)	1572 (45.5)		725 (46.7)	
Other/unknown	5556 (11.2)	4947 (10.7)	609 (17.6)		194 (12.5)	

*CrCl*, creatinine clearance; *TIA*, transient ischemic attack; *DDI*, drug-drug interaction; *EP*, electrophysiologist; *NSAIDs*, nonsteroidal anti-inflammatory drugs; *SD*, standard deviation

**Figure 1:**
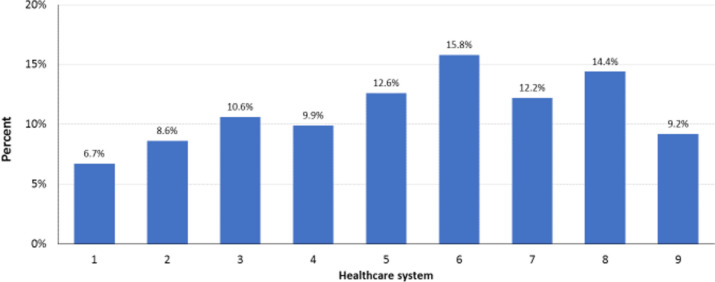
Rate of label-inconsistent dosing of direct oral anticoagulants among the healthcare systems. *P* < 0.001 for overall comparison of label-inconsistent dosing.

Of the 51,128 evaluated patients, 5008 (9.8%) were prescribed label-inconsistent doses (6.8% lower and 3.0% higher than label). Compared with label-consistent doses, the mean age and the proportion of females were statistically significantly higher among patients prescribed doses lower or higher than label, and race was significantly different among those with doses higher than label (all *P* < 0.001) (Table 1). Most clinical variables showed statistically significant differences vs label-consistent dosing; alcohol use and DDI were not significantly different for lower doses, and diabetes, liver disease, and medications predisposing to bleeding were not significantly different for higher doses (Table 1). Label-inconsistent dosing varied by healthcare system and ranged from 6.7 to 15.8% (*P* < 0.001) (Fig. 1).

### Remove extra indent Dosing Patterns by DOAC and Creatinine Clearance Categories

Label-consistent reduced doses were prescribed to 8.5% of patients overall, and between 1.4 and 9.5% of patients across the individual DOACs. The highest proportion of label-inconsistent dosing was associated with rivaroxaban (13.2%), followed by apixaban (8.7%) and dabigatran (7.2%) (Table 2); edoxaban was prescribed to only 14 patients and was not further evaluated.

**Table 2:** Dosing Patterns by Direct-Acting Oral Anticoagulant (DOAC) and Creatinine Clearance (CrCl) Categories*

Dosing strategy	Number (%)^†^			
	**Any DOAC**^**††**^	**Apixaban**	**Dabigatran**	**Rivaroxaban**
Overall	*N* = 51,128	*N* = 38,192	*N* = 592	*N* = 12,330
Label-consistent full dose	41,773 (81.7)	31,700 (83.0)	541 (91.4)	9528 (77.3)
Label-consistent reduced dose	4347 (8.5)	3159 (8.3)	8 (1.4)	1174 (9.5)
Label-inconsistent low dose	3454 (6.8)	2496 (6.5)	41 (6.9)	913 (7.4)
Label-inconsistent high dose	1554 (3.0)	837 (2.2)	2 (0.3)	715 (5.8)
Normal renal function (CrCl ≥ 60 ml/min)	*N* = 34,638	*N* = 25,079	*N* = 434	*N* = 9121
Label-consistent full dose	32,939 (95.1)	23,948 (95.5)	419 (96.5)	8569 (94.0)
Label-consistent reduced dose	142 (0.4)	142 (0.6)	0	0
Label-inconsistent low dose	1359 (3.9)	821 (3.3)	15 (3.5)	522 (5.7)
Label-inconsistent high dose	198 (0.6)	168 (0.7)	0	30 (0.3)
Moderate renal impairment (CrCl 30–59 ml/min)	*N* = 14,374	*N* = 11,254	*N* = 149	*N* = 2961
Label-consistent full dose	8480 (59.0)	7398 (65.7)	122 (81.9)	959 (32.4)
Label-consistent reduced dose	2870 (20.0)	1889 (16.8)	0	975 (32.9)
Label-inconsistent low dose	1844 (12.8)	1438 (12.8)	26 (17.5)	377 (12.7)
Label-inconsistent high dose	1180 (8.2)	529 (4.7)	1 (0.7)	650 (22.0)
Severe renal impairment (CrCl < 30 ml/min)	*N* = 2116	*N* = 1859	*N* = 9	*N* = 248
Label-consistent full dose	354 (16.7)	354 (19.0)	0	0
Label-consistent reduced dose	1335 (63.1)	1128 (60.7)	8 (88.9)	199 (80.2)
Label-inconsistent low dose	251 (11.9)	237 (12.8)	0	14 (5.7)
Label-inconsistent high dose	176 (8.3)	140 (7.5)	1 (11.1)	35 (14.1)

*Consistent and inconsistent dosing based on labels of the individual drugs

^†^Percentages may not equal 100% due to rounding

^††^Edoxaban not shown but included in any DOAC results; contributed 14 of 51,128 patients (0.03%)

When stratified by CrCl alone, higher proportions of patients with renal impairment were prescribed doses lower than label (11.9–12.8%) relative to patients with normal renal function (3.9%), and approximately 8% of patients with renal impairment were prescribed doses higher than label (Table 2). Label-inconsistent dosing was observed in 7.2–13.2% of patients prescribed each of the DOACs, and dosing lower than label was generally more prevalent than dosing higher than label with the notable exceptions of patients with moderate (CrCl 30–59 ml/min) or severe (CrCl < 30 ml/min) renal impairment taking rivaroxaban (Table 2).

### Dosing Lower than Label

**Figure 2:**
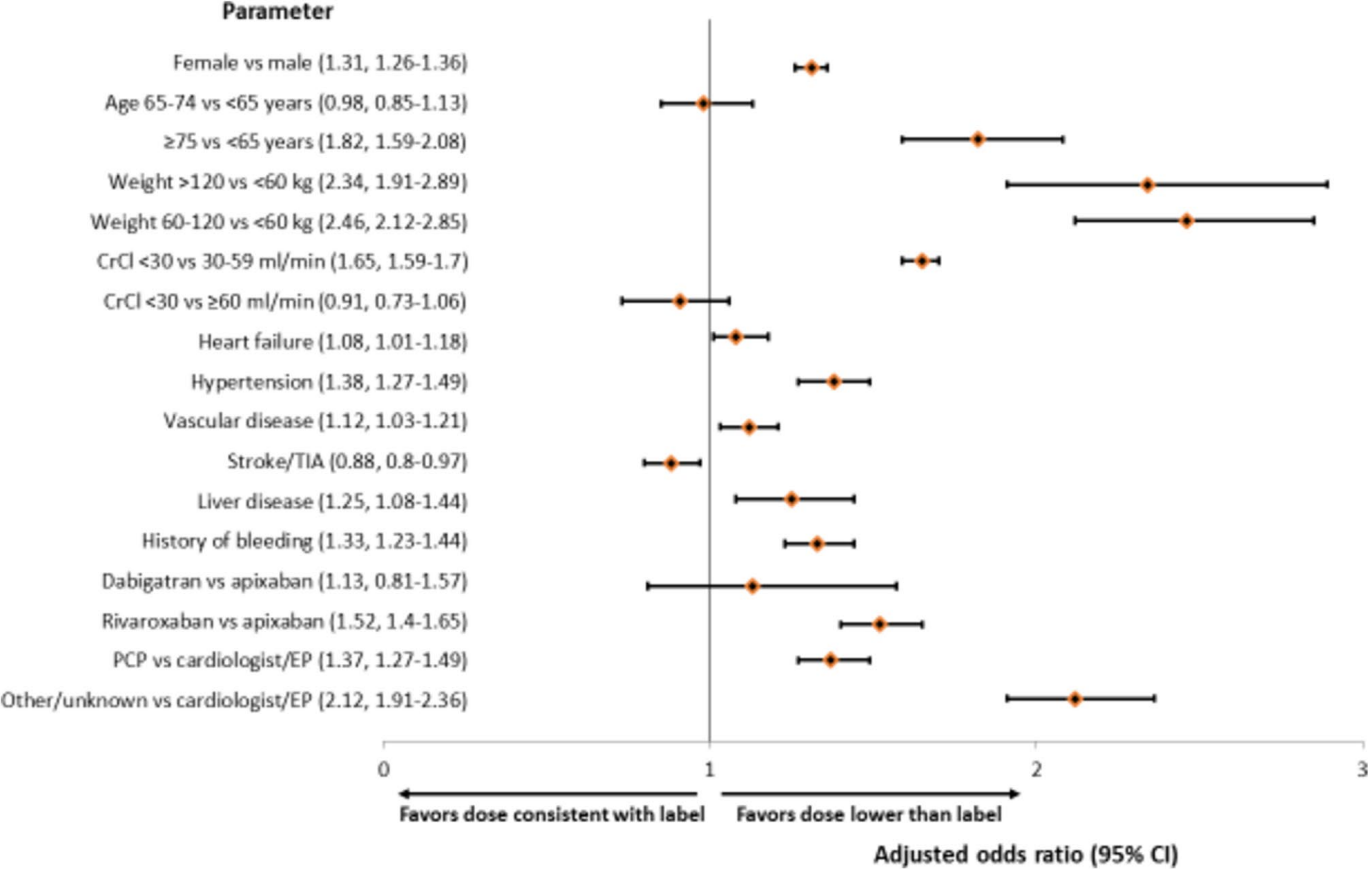
Multivariable logistical regression to identify factors associated with dosing lower than label (*N* = 3454) vs label-consistent dosing (*N* = 46,120) of direct oral anticoagulants. CI, confidence interval; CrCl, creatinine clearance; EP, electrophysiologist; PCP, primary care provider; TIA, transient ischemic attack.

Multivariable regression analysis showed that female sex, age ≥ 75 years, and higher weight were associated with a significantly higher likelihood of dosing lower than label (Fig. 2). The aOR of 1.65 and 95% CI 1.59–1.70 indicated that severe renal impairment (CrCl < 30 ml/min) was 65% more likely to be dosed lower than label compared with moderate renal impairment (CrCl 30–59 ml/min). Patients with HF, hypertension, vascular disease, liver disease, or a history of bleeding were significantly more likely to be prescribed a dose lower than label (Fig. 2). In contrast, prior stroke/TIA favored label-consistent dosing (aOR 0.88, 95% CI 0.80–0.97).

Among DOACs, rivaroxaban was 52% more likely than apixaban to have lower dosing (aOR 1.52, 95% CI 1.4–1.65) (Fig. 2). Compared with cardiologists/EPs, PCPs (aOR 1.37, 95% CI 1.27–1.49) or other/unknown clinician specialties (aOR 2.12, 95% CI 1.91–2.36) were more likely to prescribe DOACs at doses lower than label (Fig. 2).

### Dosing Higher than Label

**Figure 3:**
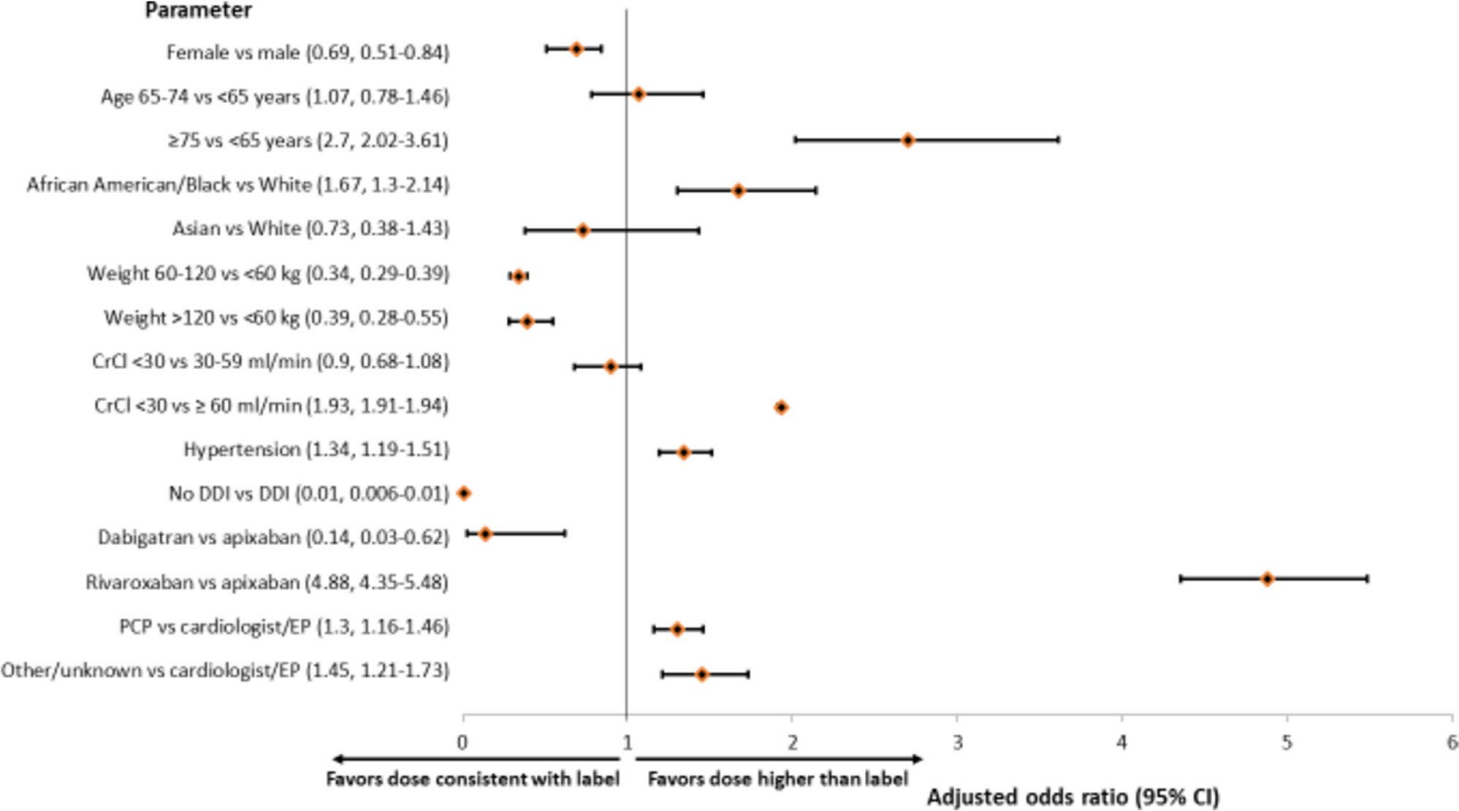
Multivariable logistical regression to identify factors associated with dosing higher than label (*N* = 1154) vs label-consistent dosing (*N* = 46,120) of direct oral anticoagulants. CI, confidence interval; CrCl, creatinine clearance; DDI, drug-drug interactions; EP, electrophysiologist; PCP, primary care provider; TIA, transient ischemic attack.

Male sex, age ≥ 75 years, and African American/Black race were all associated with a statistically significantly higher likelihood of dosing higher than label (Fig. 3). Clinical variables that significantly favored dosing higher than label were hypertension and use of drugs with potential DDIs. Weight categories of 60–120 kg and > 120 kg both favored label-consistent dosing vs weight < 60 kg. Severe renal impairment was associated with an almost two-fold higher likelihood of receiving a dose higher than label than normal renal function (aOR 1.93, 95% CI 1.91–1.94) (Fig. 3).

Use of dabigatran significantly favored label-consistent dosing vs apixaban, whereas rivaroxaban favored dosing higher than label vs apixaban and dabigatran, although the latter comparison had a very wide 95% CI (Fig. 3). Compared with cardiologists/EPs, dosing higher than label was 30% and 45% more likely when prescribed by PCPs or other/unknown clinician specialties, respectively (Fig. 3).

## DISCUSSION

In this large, real-world retrospective study, label-inconsistent dosing of DOACs was observed in 9.8% of patients with NVAF, which is lower than in a large US claims-based analysis (17.3%).^29^ As our study relied on more recent data (2022 vs 2015 to 2017), it is possible that the lower proportion reflects increasing awareness of and adherence over time to dosing recommendations among the participating healthcare systems; for example, reductions in label-inconsistent dosing over time have been reported in data from the US Veterans Health Administration and a patient registry.^27,30^ The results may also reflect the cross-sectional nature of our study, as DOAC doses were not necessarily initial prescriptions. Nevertheless, a rate of one in ten patients may be clinically relevant, as accumulating evidence suggests dosing lower or higher than label may be associated with adverse outcomes.^29,31–39^

Several variables were associated with doses both lower and higher than label, suggesting that these variables (age ≥ 75 years, hypertension, renal impairment, and prescribing by a PCP) may be indicators for label-inconsistent dosing that provide a target for anticoagulation stewardship efforts, including educating clinicians on recent prescribing recommendations.^3^ As in other studies,^18–30^ dosing lower than label was more prevalent than dosing higher than label, and may be an intentional strategy reflecting the clinician’s and/or patient’s desire to maintain anticoagulation while reducing bleeding complications,^45,46^ especially for variables that may already convey an increased risk of bleeding (e.g., age ≥ 75 years, hypertension, renal impairment). Supporting this interpretation is the observation that several HAS-BLED risk components, including a history of bleeding, significantly increased the likelihood of low label-inconsistent dosing; other studies have also reported that dosing lower than label is more likely in patients with a history of bleeding or anemia.^29,37,47–49^ Lower than label dosing strategies have not been associated with the intended effect of reducing bleeding risk and, in fact, may increase the risk of other adverse outcomes.^29,31–39^ While we could not additionally explain the association of these variables with overdosing, as our study was not designed to solicit or understand the basis for observed relationships, further evaluation of overdosing is warranted, as it is consistently reported in studies of DOACs, albeit at a lower rate than underdosing. It should also be noted that, as highlighted in recent guidelines,^3^ because some variables can change over time, DOAC dose and risk of stroke and bleeding should be re-assessed at periodic intervals as should the net clinical benefit of treatment.

Label-consistent reductions in dosing are primarily based on the presence of renal impairment, although apixaban dose reductions incorporate several factors including age, body weight, and Scr thresholds in NVAF patients rather than CrCl. However, renal impairment is also associated with dosing lower than label,^29,37^ and we observed a similar pattern, as patients with severe renal impairment had a higher likelihood of dosing lower than label vs those with moderate impairment. Renal impairment has been associated with dosing higher than label,^47,48^ which may increase bleeding risk.^31–33,37^ Those findings were replicated in our study, where approximately 8% of patients with moderate or severe renal impairment had dosing higher than label. These results emphasize the importance of accurately evaluating renal function according to both treatment recommendations^3^ and FDA-approved labeling.^14–17^ Notably, 11.3% of the original patient cohort was excluded from our analysis because of missing data for calculating Scr or CrCl rates to determine appropriate dosing. This may present a potential opportunity for initial intervention to improve NVAF quality of care by fully capturing clinical information in the health record by documenting the clinical information needed to dose the patient consistent with label.

Two variables related to clinical settings were associated with label-inconsistent dosing: clinician specialty and healthcare system. In particular, PCPs were significantly more likely to prescribe label-inconsistent doses than cardiologists/EPs. Other studies have also noted differences among specialties,^29,50,51^ suggesting heterogeneity among clinicians in their understanding of DOAC dosing recommendations and emphasizing the importance of documenting clinician rationale when making DOAC dosing decisions that are inconsistent with label. As site variation in dosing has previously been reported,^27,30,51^ it was not surprising that label-inconsistent dosing varied among the participating healthcare systems. While the reason for this observation was not determined, it may be proposed that there are inherent differences in approaches to monitoring dosing across healthcare systems, including greater familiarity with the drugs due to more prescribing by larger healthcare systems, which may also have more resources to implement consistent mitigation strategies for label-inconsistent dosing, including anticoagulation stewardship programs. However, these proposals require further investigation.

These results indicate a remaining need to improve care and monitoring of patients with NVAF prescribed DOACs. Our identification of clinical variables associated with label-inconsistent dosing provides a potential list of patient attributes that may assist healthcare systems in preventing patients from falling through the gaps of benefit-risk assessment when making dosing decisions. Incorporating these variables into resources and programs aimed at stratifying risk and monitoring treatment decisions may help close these gaps. A variety of programs have been suggested including anticoagulation stewardship, pharmacist-led monitoring programs, anticoagulation services, EHR-based clinical decision support, and quality care/improvement initiatives including population health digital dashboards, which can enhance tracking of quality care performance metrics.^12,51,52^ Wider implementation of all such programs will likely be required to achieve the overall goal of improvement in patient outcomes.

### Limitations

Study limitations include incomplete data, incorrectly coded data, and unavailability of data from healthcare utilization outside the participating healthcare systems’ EHRs. Another limitation is that clinical outcomes were not captured. This study was limited to healthcare systems that consented to participate, potentially introducing selection bias, as these systems may already have or may be more amenable to programs for enhancing appropriate DOAC use. In this regard, selection of a dose other than that recommended in product labeling may reflect clinical considerations that cannot be fully captured in a retrospective manner. Thus, the results are only applicable to the population studied and may not be generalizable to other populations.

## CONCLUSIONS

Label-inconsistent dosing of DOACs in patients with NVAF was 9.8%, which may be underestimated as 11% of patients did not have sufficient clinical data to assess label consistency. Label-inconsistent dosing was primarily lower than label and was observed in up to 15.8% of patients depending on the healthcare system. These results suggest opportunities to improve DOAC prescribing. Identification of clinical variables associated with dosing lower and higher than label may suggest approaches for enhancing evaluation of patient risk factors to ensure appropriate dosing. Similarly, differences in clinician specialties that may be predictive of label-inconsistent dosing, as well as observed variations in dosing among healthcare systems, highlight the need for allocation of resources to support programs that better evaluate the risks and needs of patients with NVAF receiving DOACs.

## Supplementary Information

Below is the link to the electronic supplementary material.

Supplementary file1 (DOCX 21 KB)
