# Supplementary material for: Direct-Acting Oral Anticoagulants and Potential Inconsistencies with FDA-Approved Dosing for Non-Valvular Atrial Fibrillation: A Retrospective Real-World Analysis Across Nine US Healthcare Systems
Source: J Gen Intern Med. 2024 Oct 18;40(4):828–37. doi: 10.1007/s11606-024-09106-w (PMC11914546; doi:10.1007/s11606-024-09106-w)
Supplement: Supplementary file 1 — Supplementary file1 (DOCX 21 KB) [file 11606_2024_9106_MOESM1_ESM.docx]

Supplementary Information

**Supplemental Appendix Table 1** Atrial Fibrillation Diagnosis Codes

| **ICD-10 code** | **Description** |
| --- | --- |
| I48 | Atrial fibrillation and flutter |
| I480 | Paroxysmal atrial fibrillation |
| I481 | Persistent atrial fibrillation |
| I4811 | Longstanding persistent atrial fibrillation |
| I4819 | Other persistent atrial fibrillation |
| I482 | Chronic atrial fibrillation |
| I4820 | Chronic atrial fibrillation, unspecified |
| I4821 | Permanent atrial fibrillation |
| I483 | Typical atrial flutter |
| I484 | Atypical atrial flutter |
| I489 | Unspecified atrial fibrillation and atrial flutter |
| I4891 | Unspecified atrial fibrillation |
| I4892 | Unspecified atrial flutter |

**Supplemental Appendix Table 2** United States Food and Drug Administration Approved Dosing

| **Drug** | **Standard dosing** | **Renal adjustment** | **Drug-drug interactions** |
| --- | --- | --- | --- |
| Apixaban (Eliquis®)^14^ | 5 mg twice daily | - 2.5 mg twice daily if at least 2:   - Age ≥80 years   - Body weight ≤60 kg   - Scr ≥1.5 mg/dL | - 2.5 mg twice daily when coadministered with drugs that are combined P-glycoprotein and strong cytochrome P450 3A4 inhibitors |
| Dabigatran (Pradaxa®)^15^ | 150 mg twice daily | - 75 mg twice daily if CrCl 15–30 mL/min - Avoid if CrCl <15 mL/min or on hemodialysis) | - 75 mg twice daily if given with P-glycoprotein inhibitors, dronedarone, or systemic ketoconazole |
| Edoxaban (Savaysa®)^16^ | 1. mg once daily | - 30 mg once daily if CrCl 15–50 mL/min - Avoid if CrCl >95 mL/min or <15 mL/min | - No dose reduction is recommended for concomitant P-glycoprotein inhibitor use |
| Rivaroxaban (Xarelto®)^17^ | 20 mg once daily with evening meal | - 15 mg once daily with evening meal if CrCl <50 mL/min or if on hemodialysis | - Avoid combined P-glycoprotein and strong cytochrome P450 3A4 |

***Abbreviations:*** *CrCl creatinine clearance, Scr serum creatinine*

**Supplemental Appendix Table 3** Characteristics of Participating Healthcare Systems (*N*=9)

| **Attribute** | **Number of healthcare systems** |
| --- | --- |
| Geographic region |  |
| Northeast | 2 |
| Midwest | 7 |
| Academic affiliation | 6 |
| DSH/FQHC/SafetyNet Hospital | 3 |
| Dosing quality initiative* | 8 |
| Inpatient only | 3 |
| Outpatient only | 1 |
| Both inpatient and outpatient | 4 |
| Anticoagulation services | 9 |
| Warfarin only | 3 |
| All oral anticoagulants | 6 |
| Anticoagulation stewardship | 5 |

***Abbreviations:*** *DSH, Disproportionate Share Hospital, FQHC Federally Qualified Health Center*

*Each site determined if their services qualified as a dosing quality initiative.
